# Comparative Studies on Regioselectivity of α- and β-Linked Glucan Tosylation

**DOI:** 10.3390/molecules25225382

**Published:** 2020-11-17

**Authors:** Andreas Koschella, Thomas Heinze, Antje Tied, Katja Geitel, Chih-Ying Chien, Tadahisa Iwata

**Affiliations:** 1 Institute for Organic Chemistry and Macromolecular Chemistry, Friedrich Schiller University of Jena, Humboldtstraße 10, D-07743 Jena, Germany; andreas.koschella@uni-jena.de (A.K.); antje.tied@uni-jena.de (A.T.); katja.geitel@uni-jena.de (K.G.); f80608@icloud.com (C.-Y.C.); 2 Science of Polymeric Materials, Department of Biomaterial Sciences, Graduate School of Agricultural and Life Sciences, The University of Tokyo, 1-1-1 Yayoi, Bunkyo-ku, Tokyo 113-8657, Japan; atiwata@mail.ecc.u-tokyo.ac.jp

**Keywords:** α-1,3-glucan, β-1,3-glucan, medium-controlled selectivity, NMR spectroscopy, regioselectivity, tosylation

## Abstract

Alpha- and beta-linked 1,3-glucans have been subjected to conversion with *p*-toluenesulfonic acid (tosyl) chloride and triethylamine under homogeneous reaction conditions in *N*,*N*-dimethyl acetamide/LiCl. Samples with a degree of substitution of tosyl groups (DS_Ts_) of up to 1.91 were prepared by applying 5 mol reagent per mole repeating unit. Hence, the reactivity of α-1,3-glucan is comparable with cellulose and starch, while the β-1,3-linked glucan curdlan is less reactive. The samples dissolve in aprotic dipolar media independent of the DS_Ts_ and possess a solubility in less polar solvents that depends on the DS_Ts_. NMR studies on the tosyl glucans and of the peracylated derivatives showed a preferred tosylation of position 2 of the repeating unit. However, the selectivity is less pronounced compared with starch. It could be concluded that the α-configurated glycosidic bond directs tosyl groups towards position 2.

## 1. Introduction

Conversion of polysaccharides may lead to products with different functionalization patterns. In most cases, the order of reactivity is *O*-6 >> *O*-2 > *O*-3 provided that the steric demand of the reagent is low and that the conversion proceeds under homogeneous reaction conditions.

A preferred or even exclusive reaction at the primary hydroxyl group is observed in the case of a high steric demand of the reagent. Typical examples are the well-known protecting group reagents triphenylchloromethane [1] and bulky trialkylchlorosilanes [2,3]. Regioselective esterification of cellulose could not yet be achieved [4].

An interesting observation was made during the transesterification of starch dissolved in dimethyl sulfoxide with carboxylic acid vinyl esters in the presence of inorganic salts. It could be shown that weak acidic or basic as well as neutral salts induce exclusive esterification of position 2 of starch without using of laborious protecting group techniques [5]. This impressive regioselectivity is explained with the presence of α-1,4-glycosidic bonds instead of β-1,4-glycosidic bonds that occur in cellulose. This concept of medium-controlled selectivity has been proofed because *O*-2-acylation could be accomplished for both nigeran and pullulan as well [5]. This selectivity is not limited to carboxylic acid esters. It was observed that the tosylation of polysaccharides may also yield regioselective conversion depending on the structure of the polymer backbone, i.e., α- or β-linkages. The tosylation of cellulose dissolved in the non-derivatizing cellulose solvent *N*,*N*-dimethyl acetamide (DMA)/LiCl [6] leads to a preferred tosylation of *O*-6 [7]. On the contrary, the conversion of starch dissolved in DMA/LiCl with *p*-toluenesulfonic acid chloride (TsCl) in the presence of triethylamine leads to a product with unexpected selective tosylation of position *O*-2 [8]. Thus, the α-glycosidic linkage in combination with the solvent used may influence the regioselectivity of the chemical reaction. Obviously, the accessibility of hydroxyl groups is limited by a certain (e.g., helical) conformation of the polymer in solution [9,10,11,12]. Tosylation studies on lichenan and pullulan exhibited a lower reactivity compared with cellulose but preferred tosylation of the primary hydroxyl group [13]. In particular, Ts cellulose has been found as a platform compound for the synthesis of further derivatives. They can impart solubility or the remaining hydroxyl groups are subsequently functionalized. A more sophisticated reason is that the Ts group acts as a leaving group in nucleophilic displacement reactions [14].

In the course of our ongoing research, a novel biosynthetically produced α-1,3-linked glucan came in the center of our interest [15,16]. Our own results on acetylation of this polysaccharide under homogeneous reaction conditions in DMA/LiCl revealed a pronounced acylation of position 6, followed by position 2 [17]. Position 4 exhibited the lowest reactivity.

Due to its α-glycosidic linkage, it is worth studying the selectivity of the tosylation reaction in comparison with curdlan having β-1,3-linkages. In the present paper, we wish to report on the tosylation of α-1,3-glucan considering the maximum degree of substitution and the functionalization pattern of the product.

## 2. Results

### 2.1. Synthesis

**Figure 1:**
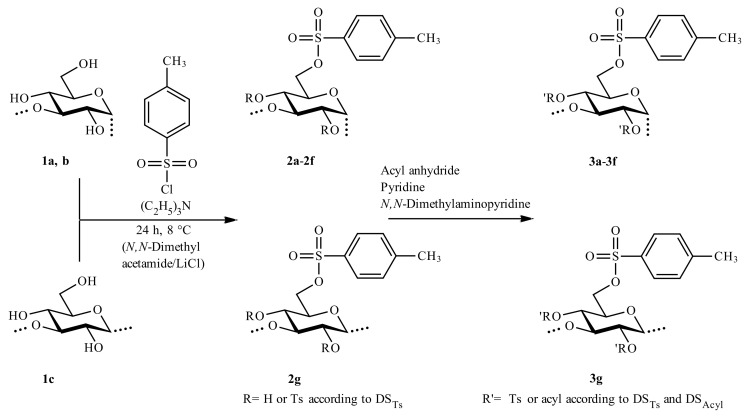
Synthesis of 1,3-glucan *p*-toluenesulfonic (Ts) acid esters by conversion of the biopolymer dissolved in *N*,*N*-dimethyl acetamide/LiCl with *p*-toluenesulfonic acid chloride and triethylamine was followed by peracylation (degree of substitution of tosyl groups, DS_Ts_, of acyl groups, DS_Acyl_).

The tosylation of the glucans **1a–c** was conducted under homogeneous conditions by dissolving the polysaccharides in DMA/LiCl followed by addition of triethylamine and TsCl. After 24 h at 8 °C, the polymer was precipitated, collected, and analyzed after washing and drying. Peracylation introduced a probe for the determination of the functionalization pattern by ^1^H-NMR spectroscopy (Figure 1). The molar ratio of TsCl:anhydroglucose unit (AGU) was varied (Table 1).

Conversion of 1 mol **1a** with 1 mol TsCl afforded a tosyl glucan with a degree of substitution of Ts groups (DS_Ts_) 0.63 (sample **2a**). The DS_Ts_ of the tosyl glucan increases with the increasing molar ratio of TsCl:AGU. It is noticed that the reagent efficiency is 63–65% up to a molar ratio of TsCl:AGU of 2:1. A further increase of the molar ratio leads to increased DS_Ts_; however, the reagent efficacy became lower (e.g., sample **2d**, 48% and sample **2e**, 38%), a behavior that is not unexpected. The highest DS_Ts_ obtained was 1.91 applying 5 mol TsCl per mole AGU (sample **2e**). Thus, the reactivity of glucan **1a** is comparable with starch, where a conversion with 6 mol TsCl per mol AGU led to a product with DS_Ts_ 2.02 [8] as well as to cellulose giving a product with DS_Ts_ 2.02 under comparable conditions [7]. The degree of polymerization (number averaged, DP_n_ and weight averaged, DP_w_) of the starting material does not influence the DS_Ts_ obtained significantly. Compare samples **2b** (from **1a**, DS_Ts_ 1.00) and **2f** (from **1b**, DS_Ts_ 0.96).

### 2.2. Properties

The solubility of glucan tosylates in organic solvents of different polarity depends on their DS_Ts_ (Table 1). However, the samples dissolve in DMA, *N*,*N*-dimethyl formamide, and dimethyl sulfoxide (DMSO) independent of the DS_Ts_. Starting at DS_Ts_ 1.00, the samples dissolve in THF as well (sample **2b**). A further increase of the DS_Ts_ to ≥1.3 yielded acetone-soluble samples (samples **2c**, **2d**, **2e**). Samples with DS_Ts_ > 1.45 are soluble in chloroform as well (**2d**, **2e**). The solubility of the glucan derivatives is comparable with the solubility of tosyl cellulose [7]. The only difference is that the solubility limit is approximately 0.3 DS_Ts_ units lower in case of the 1,3-glucans.

### 2.3. Structure Characterization

**Figure 2:**
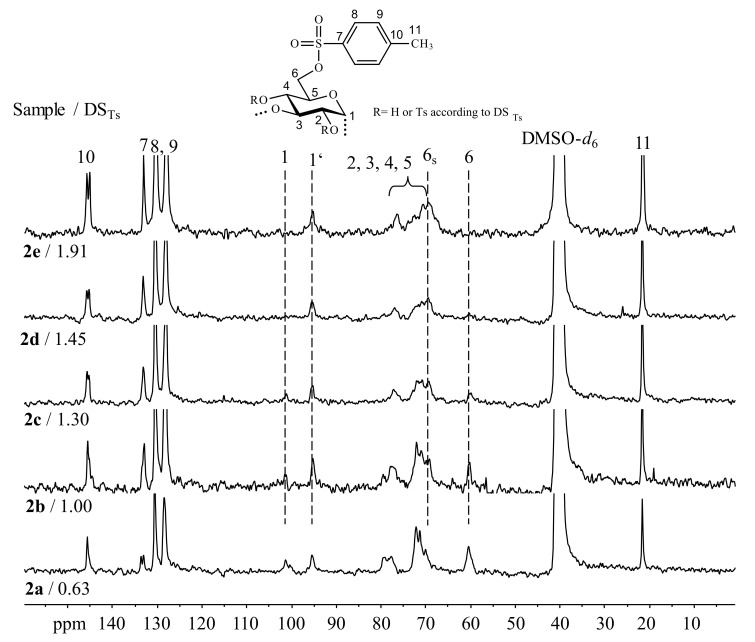
^13^C-NMR spectra of α-1,3-glucan *p*-toluenesulfonic (Ts) acid esters of different degrees of substitution of Ts groups (DS_Ts_) recorded in dimethyl sulfoxide-*d*_6_ (DMSO-*d*_6_). Index s means functionalized position. Dash (‘) means influenced by functionalization of the neighboring position.

The tosylated α-1,3-glucans were subjected to ^13^C-NMR spectroscopy in order to gain information on the functionalization pattern of the Ts groups within the AGU (Figure 2).

The ^13^C-NMR spectrum of sample **2a** (DS_Ts_ 0.63) shows all the signals of the polymer backbone and the expected structural features. The signals of the Ts moiety are detected at 21.6 ppm (methyl group, C-11) as well as at 128.4 ppm and 130.5 ppm (aromatics, C-8,9), 133.0 ppm and 133.5 ppm (aromatics, C-7), and 145.4 ppm (aromatics, C-10). The signal of the CH_2_OH-group (C-6) appears at a chemical shift of 60.3 ppm, while the peak of the CH_2_-OTs can be found at 69.9 ppm. The carbon atoms of positions 2, 4, and 5 lead to resonances around 71–72 ppm. Position 3 is expected to give the signal between 77.4 and 79.5 ppm. The signal for position 1 is split off in two signals appearing at 95.3 ppm (C-1’) and at 101.3 ppm (C-1). This is caused by partial tosylation of position 2 of the AGU. It must be mentioned that this is observed already at low DS_Ts_ of 0.63. The signal for position 6 is split as well, which is caused by DS_Ts_ < 1. Moreover, the intensity signal for C-1 is decreasing with increasing DS_Ts_. At DS_Ts_ 1.45 (sample **2d**), it disappeared completely, which indicated a complete tosylation of position 2. However, the resolution of the ^13^C-NMR spectra is poor, which can be explained with the rigid structure of the dissolved polymer.

In order to get a deeper insight into the distribution of Ts groups within the repeating unit, acylation of the tosylated polysaccharide derivatives was performed in order to introduce an NMR-probe. It is known that both the carbonyl carbons and the alkyl carbon atoms of carboxylic acid esters show a chemical shift that depends on its position within the repeating unit. Hence, partial functionalization of position 2, 4, and 6 can be elucidated.

**Figure 3:**
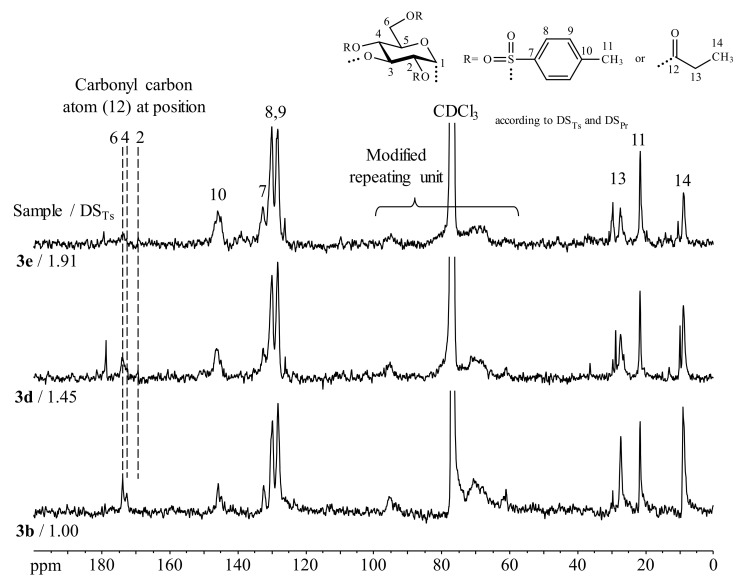
^13^C-NMR spectra of perpropionylated α-1,3-glucan *p*-toluenesulfonic acid esters of different degrees of substitution (of tosyl groups, DS_Ts_, of propionyl groups, DS_Pr_) recorded in CDCl_3_.

Studies on peracetylation of tosyl glucan samples turned out that peracetylation is achieved but the samples do not dissolve to a sufficient extent. Perpropionylated samples sufficiently dissolve in CDCl_3_ for NMR spectroscopy. The resolution of the ^1^H-NMR spectra is poor despite the peracetylation performed. Thus, calculation of both DS_Ts_ and the degree of substitution of propionyl groups (DS_Pr_) from those data is defective. However, conclusions regarding the functionalization pattern could be drawn from the ^13^C-NMR spectra (Figure 3).

In the case of **3f** and **3b** (DS_Ts_ around 1.00), two carbonyl carbon peaks at chemical shifts of 173.9 ppm and 172.4 ppm have been observed, which can be assigned as propionyl moieties attached to positions 4 and 6 of the modified repeating unit. A further peak at 169.6 ppm, which is related to a propionyl group at position 2, cannot be seen. This suggests a Ts group on position 2 position only. Therefore, it can be assumed that the Ts group has been regioselectively introduced at position 2 of the α-1,3-glucan.

**Figure 4:**
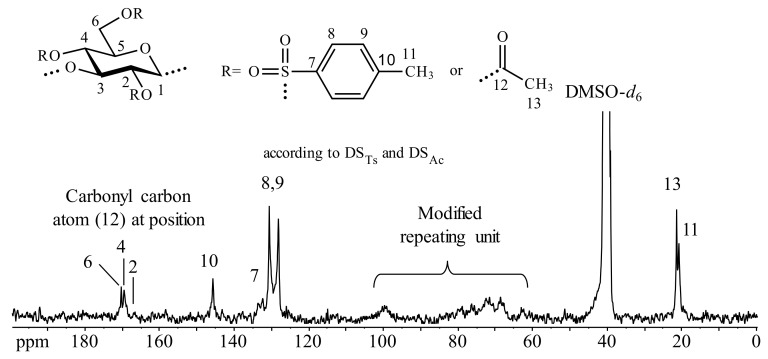
^13^C-NMR spectrum of peracetylated β-1,3-glucan *p*-toluenesulfonic acid ester (sample **3g**) degree of substitution (of tosyl groups, DS_Ts_, 1.02, of acetyl groups, DS_Ac_) recorded in dimethyl sulfoxide (DMSO)-*d*_6_.

The ^13^C-NMR spectrum of the peracetylated tosyl β-1,3-glucan (starting from curdlan **1c**, sample **3g**, Figure 4) looks similar to those of the α-1,3-glucan derivatives. Thus, peaks of high intensity appear that are attributed to carbonyl carbon atoms bound to the positions 6 (170.3 ppm) and 4 (169.5 ppm). However, a signal at 166.9 ppm indicates a slight acetylation of position 2, while no signal of acylated position 2 could be detected in the case of tosyl α-1,3-glucan of comparable DS_Ts_ (sample **3b**, Figure 3). This finding suggested that the regioselectivity of the tosylation reaction is influenced by the configuration of the anomeric carbon atom also in the case of 1,3-linked polysaccharides but to a smaller extent compared with 1,4-linked polysaccharides cellulose and starch.

## 3. Discussion

A distinct influence on the linkage type on the reactivity could be detected. Comparison of the differently linked polysaccharides revealed that the β-1,3-linked polymer **1c** was found to be less reactive (DS_Ts_ 1.02, sample **2g**) compared with the α-1,3-linked polymer **1a** (DS_Ts_ 1.30, sample **2c**). Obviously, the type of glycosidic linkage influences the reactivity. The introduction of 6-deoxy-6-chloro moieties, which is a common side reaction, is negligible, i.e., the degree of substitution of 6-deoxy-6-chloro groups (DS_Cl_) is ≤ 0.03 for all samples.

The solubility of the polysaccharide derivatives is influenced by the type of substituent, the DS, the functionalization pattern within the repeating unit and along the polymer chain as well as by the linkage type of the polymer backbone. Obviously, the influence of the linkage structure does not affect the solubility of the polysaccharide tosylates significantly. The slightly lower DS_Ts_, where solubility in a certain solvent is observed, is not considered to be significant. One can conclude that the solubility of the different polysaccharides at comparable DS_Ts_ is also comparable.

In contrast, the regioselectivity of tosylation is of major importance for subsequent reactions, in particular, nucleophilic displacement reactions. As demonstrated for cellulose, nucleophilic displacement reactions with amines yield derivatives bearing 6-deoxy-6-amino groups. Such compounds have found various applications due to their ability to form monolayers or nanoparticles, which can further be modified by functionalization of the amino groups [18]. It is known that Ts groups bound to secondary positions are hard to displace compared with primary Ts groups. It was found that the α-1,3-glucan **1** is tosylated at position 2 with a certain preference. Therefore, subsequent nucleophilic displacement reactions are expected to run with low efficiency. This must be considered for starch and the biotechnologically produced glucan **1**.

## 4. Materials and Methods

### 4.1. Materials

The α-1,3-glucan was provided by the DuPont company (Wilmington, DE, US, **1a**, DP_n_ 269, DP_w_ 1261, **1b** DP_n_ 421, DP_w_ 2635) and dried for in vacuum for 2 d over potassium hydroxide. Curdlan (Megazyme u.c., Wicklow, Ireland, **1c**) was dried in the same way. Lithium chloride (Honeywell) was dried in vacuum at 150 °C prior to use. TsCl and DMA (Acros Organics) were used as received. Triethylamine has been distilled from calcium hydride in order to remove moisture.

### 4.2. Tosylation of α-1,3-glucan 1a (Sample ***2c***)

α-1,3-Glucan **1a** (5.0 g, 0.0308 mol) was slurried in 100 mL DMA under exclusion of moisture and stirred for 2 h at 120 °C. After cooling to 100 °C, LiCl (7.5 g) was added and stirring was continued until complete dissolution of the polymer. A mixture of 17 mL (0.121 mol, 4 mol/mol AGU) triethylamine and 17 mL DMA was added dropwise while the mixture was cooled to 8 °C before TsCl (11.8 g, 0.062 mol, 2 mol/mol, AGU) was added. The mixture was allowed to react for 24 h at 8 °C under stirring before the polymer was precipitated in 500 mL of ice water. The precipitate was collected by filtration, washed with water (2 times, 200 mL) and ethanol (3 times, 200 mL) and finally dried in vacuum at 60 °C.

Yield: 10.13 g (91%).

DS_Ts_ = 1.30, DS_Cl_ = 0.03 (calculated from sulfur respectively chlorine content from elemental analysis).

Elemental analysis: C 49.94, H 4.95, S 11.47, Cl 0.33

IR (KBr): 3540 ν(OH), 3066 ν(=CH), 2955 ν(CH_2_), 1598 ν(C = C_aromat_), 1495, 1451 δ(CH_2_, CH_3_), 1361 δ(CH_3_), ν_as_(SO_2_), 1191 ν(C–O–C), 1177 ν_s_(SO_2_), 1058 ν(C–O–C), 813 δ(=C-H_aromat_) cm^−1^.

^13^C-NMR (DMSO-*d*_6_): 145.6, 145.1, 133.1, 130.5, 127.9 (C_aromat_), 95.6 (C-1), 95.1 (C-1’), 77.2–69.2 (C-2,3,4,5), 59.8 (C-6), 21.6 ppm (Ts-CH_3_).

### 4.3. Perpropionylation of Tosyl α-1,3-glucan Sample (Sample ***3b***)

Tosyl-α-1,3-glucan **AT-TH81** (0.50 g) was dissolved in pyridine (25 mL) under stirring at 80 °C for 1 h. Propionic anhydride (25 mL) was added to the solution and the mixture was allowed to react for 24 h at 80 °C. The brownish mixture was poured into 400 mL water to precipitate the polymer. After filtration, the product was washed with water (4 times, 200 mL) and dried at 105 °C in vacuum. Perpropionylation was revealed by FTIR spectroscopy.

Yield: 0.58 g (85%).

IR (KBr): 3536 ν(OH), 2982 ν(=CH), 2945 ν(CH_2_), 1747 ν(C = O), 1598 ν(C = C_aromat_), 1495, 1463 δ(CH_2_, CH_3_), 1367 δ(CH_3_), ν_as_(SO_2_), 1247 ν(C-O), 1192 ν(C–O–C), 1179 ν_s_(SO_2_), 1058 ν(C–O–C), 813 δ(=C-H_aromat_) cm^−1^.

^13^C-NMR (CDCl_3_): 173.8, 172.7 (C-13, C=O), 145.9 (C10), 132.5 (C-7), 130.0 (C-8), 128.3 (C-9), 95.6 (C-1), 60.8–75.8 (C-2, 3, 4, 5, 6), 27.3 (C-14), 21.5 (C-11), 8.8 ppm (C-15).

### 4.4. Measurements

The Fourier transform infrared (FTIR) spectroscopy data were recorded on a Nicolet AVATAR 370 DTGS spectrometer using the potassium bromide (KBr) technique. The spectra were recorded with 64 scans in the range of 400–4000 cm^−1^ and a resolution of 4 cm^−1^. The KBr pellets were dried at 80 °C for 5 h prior to analysis.

The NMR spectra were acquired on a Bruker Avance 250 MHz and a Bruker Avance 400 MHz at 40 °C (CDCl_3_) and 60 °C (DMSO-*d*_6_) with 16 scans for ^1^H-NMR spectroscopy and up to 20,000 scans for ^13^C-NMR spectra using 60 mg sample per mL solvent.

Elemental analysis (C, H, S content) was carried out using a Vario EL III (Elementaranalysensysteme Hanau, Germany). The chlorine content was determined according to Schöniger’s method [19].

## 5. Conclusions

*p*-Toluenesulfonic acid (tosyl) esters of α-1,3-glucan could be prepared under homogeneous reaction conditions. The reactivity in terms of DS_Ts_ realized is comparable with those of cellulose and starch. However, a pronounced tosylation of the secondary hydroxyl group at position 2 was detected. This indicates that the alpha linkage of the glycosidic bond seems to be responsible for this selectivity, which could be found for starch but not for the β-1,4-linked cellulose. Even peracetylated samples exhibit a low solubility, which might be in relation with the structure of the polymer in solution. Investigations towards the macromolecular structure in the dissolved state will be carried out in the near future.

## Figures and Tables

**Table 1:** Conditions for and results of the conversion of 1,3-glucans dissolved in *N*,*N*-dimethyl acetamide/LiCl with *p*-toluenesulfonic acid chloride (TsCl) in the presence of triethylamine for 24 h at 8 °C.

Conditions		Sample	Results							
Glucan ^1^	TsCl ^2^		DS_Ts_^3^	DS_Cl_^3^	Solubility ^4^					
					DMSO	DMA	DMF	THF	Acet.	CHCl_3_
**1a**	1.0	**2a**	0.63	0.00	+	+	+	-	-	-
**1a**	1.5	**2b**	1.00	0.03	+	+	+	+	-	-
**1a**	2.0	**2c**	1.30	0.03	+	+	+	+	+	-
**1a**	3.0	**2d**	1.45	0.03	+	+	+	+	+	+
**1a**	5.0	**2e**	1.91	0.03	+	+	+	+	+	+
**1b**	1.5	**2f**	0.96	0.00	+	+	+	+	-	-
**1c**	2.0	**2g**	1.02	0.03	+	+	+	-	-	-

^1^ α-1,3-Glucan (**1a**, **1b**), β-1,3-glucan (**1c**). ^2^ Moles TsCl per mol anhydroglucose unit; 2.0 mol triethylamine per mole TsCl were applied. ^3^ Degree of substitution of Ts (DS_Ts_)- and 6-deoxy-6-chloro groups (DS_Cl_), calculated from sulfur and chlorine contents based on elemental analysis. ^4^ Dimethyl sulfoxide (DMSO), *N*,*N*-dimethyl acetamide (DMA), *N*,*N*-dimethyl formamide (DMF), tetrahydrofuran (THF), acetone (Acet.), soluble (+), insoluble (-).
